# “[CERVICAL CANCER], NOT AT THIS AGE…”: TREATMENT EXPERIENCES OF OLDER WOMEN WITH ADVANCED CERVICAL CANCER

**DOI:** 10.1093/geroni/igae098.4295

**Published:** 2024-12-31

**Authors:** Runcie Chidebe, Ifeoma Okoye

**Affiliations:** Miami University, Oxford, Ohio, United States; University of Nigeria, University of Nigeria, Enugu, Nigeria

## Abstract

**Background:**

Cervical cancer (CC) is 90% preventable with the HPV vaccine against sub-types 16 and 18, yet an estimated 21 women die every day from CC, and 7,968 die annually in Nigeria. Most of the women diagnosed with CC are older women, yet the gerontological aspect of CC among this population is largely scarce. Older women living with CC are at risk of undertreatment and are most likely to report the worst prognosis. This phenomenological study is aimed at understanding the treatment experiences of older women living with stage IV CC and their meaning-making of this incurable disease.

**Method:**

Using interpretive phenomenological analysis (IPA), 16 older women (50 – 85 years) with advanced CC were recruited. Data was collected through semi-structured interviews, and the six steps of IPA were used for analysis.

**Results:**

Four identified themes: “[cervical cancer], not at this age, not at any age anyway but at this age to me is even worse”, “He [the doctor] talked about surgery but because of my age, surgery was moved out”, “There is a need for support and to be loved” and “When someone’s [cancer] is palliative, take it as palliative, when it is critical treat it as such. Don’t treat palliative and leave critical aside”.

**Conclusion:**

This study provided a gerontological insight into the lived experiences of these older women. As the world is working to eliminate cervical cancer with the human papillomavirus vaccine, older women who already have advanced cervical cancer should not be forgotten.

